# Tuna Dark Muscle Feeding Improved the Meat Quality of Holland Mini-Piglets and Modulated the Gut Microbiota

**DOI:** 10.3390/foods13101577

**Published:** 2024-05-18

**Authors:** Chenyang Lu, Yuanming Zhang, Yang Qin, Jun Zhou, Yanbo Wang, Xiurong Su, Jiaojiao Han

**Affiliations:** 1 School of Food Science and Biotechnology, Zhejiang Gongshang University, 18 Xuezheng Road, Hangzhou 310018, China; 2 School of Marine Science, Ningbo University, 169 Qixing South Road, Ningbo 315832, China

**Keywords:** pork, tuna dark muscle, nutritional supplement, meat quality, gut microbiota

## Abstract

Pork is one of the most widely produced and consumed meats in the world, and it is also an important source of animal protein. The continuous rise in feed prices has forced the pig industry to consider adding cost-effective alternative feed to pig diets. In this study, we aimed to explore the beneficial effects of tuna dark muscle as a nutritional supplement on the growth performance, serum lipids and antioxidant levels of Holland mini-piglets, as well as on the odor and volatile substances of pork and the gut microbiota. Two-month-old male mini-piglets (*n* = 24) were fed a control diet or supplemented with either 2% (LD) or 4% (HD) tuna dark muscle for 8 weeks. The use of tuna dark muscle at low and high dosages significantly increased the average daily weight gain, but it showed no significant effect on organ indices or blood lipids. In addition, dark muscle treatment significantly increased the antioxidant capacity, characterized by increased SOD and GSH-Px activities, and it decreased the content of MDA in serum. Moreover, tuna dark muscle feeding shifted the odor of rib muscle and tendon meat away from that of the control group, while similar odor patterns were observed in the longissimus dorsi muscle. Among these volatile substances, hexanal, nonanal, and heptanal increased in response to dietary tuna dark muscle and were regarded as indispensable contributors to the feeding. Furthermore, dietary tuna dark muscle modulated the gut microbiota of the piglets, increasing the abundance of beneficial bacteria such as butyric acid-producing bacteria, and reduced the abundance of harmful bacteria. The feeding strategy reported in this study not only reduces the production costs of pork but also utilizes tuna processing by-products in an environmentally friendly way.

## 1. Introduction

Pork is one of the most widely produced and consumed meats and an important source of animal protein worldwide [1] due to its high contents of biological value protein (17.3–22.2%), fat (4.7–31.8%), and micronutrients, including iron (0.6–1.3 mg/100 g), zinc (1.6–2.7 mg/100 g), phosphorus (167–221 mg/100 g), and vitamin B12 (1 mg/100 g) [2]. Various strategies have been developed and applied to improve the growth performance of pigs and enhance pork quality, including dietary intervention, breeding, management practices, and production system optimization. Among them, dietary intervention is known as one of the most common methods [3]. An alfalfa silage diet improved the fatty acid and amino acid levels in pork [4]. Dietary whole or crushed linseed increased the n-3 PUFA content and showed no deleterious effects on the organoleptic characteristics of pork [5]. In addition, dietary seaweeds at low doses (1–2%) were reported to increase piglet productivity, pork quality, and safety [6]. However, compared with traditional plant protein, animal protein has a more similar nutritional content profile, higher production, and a lack of anti-nutritional components [7], so it might be a better alternative ingredient in piglet husbandry.

Tuna is a highly migratory deep-sea fish, and it is widely accepted worldwide due to its good nutritional and sensory quality, as well as potential health benefits [8]. During its processing, 50–70% of by-products are generated, with dark muscle accounting for a relatively high proportion [9]. The dark muscle of tuna contains rich proteins (~18%) and bioactive substances (especially active peptides), but it has coarse fibers and a poor taste, and it is not easy to process or store [9,10]. In recent years, various beneficial effects of tuna dark muscle hydrolysates on human health have been reported, including anti-hyperuricemia, blood-pressure-lowering, and cholesterol-lowering effects [11,12]. Our previous studies showed that tuna dark muscle hydrolysate regulated the Keap1 (Kelch-like ECH-Associating protein 1)-Nrf2 (nuclear factor erythroid 2 related factor 2)-ARE (antioxidant response element) pathway and showed antioxidant activity [10]. As previously reported, dietary supplementation with natural antioxidants, such as chlorogenic acid and resveratrol, improves the meat quality of pigs [13,14]. Therefore, we propose that tuna dark muscle might be a suitable alternative feed additive for piglets. Previous studies reported that the dietary tuna dark muscle enzymatic hydrolysis improved the growth performance and antioxidant activity of piglets [15], tuna fishmeal feeding increased the n-3 PUFA levels in pork [16], and dietary tuna fish oil altered the phospholipid balance and fatty acid composition of adult pigs [17]. However, the beneficial effects of whole tuna dark muscle on the growth performance of pigs and the quality of pork remain unclear.

On the one hand, the hydrolysate of tuna roe and dark muscle has been found to modulate the gut microbiota composition and increase the abundance of *Lactobacillus* and *Bifidobacterium*, as well as the production of 3-indolepropionic acid and short-chain fatty acids [15,18,19]. On the other hand, many studies have found that pork quality is closely related to the gut microbiota of pigs. A study carried out a complete longitudinal analysis of the succession of the gut microbiota of pigs throughout their lives and identified the characteristics of the gut microbiota at different growth stages and the core microbiota members, indicating that pig feeding plays an important role in the shaping of the microbiota [20]. Through deep metagenomic sequencing, a comprehensive catalog of the genes in the pig’s gut microbiota was constructed across a wide range of samples, confirming the important role of the gut microbiota in pig health and production [21].

Mini-piglet is a rare breed of small pig with tender meat and a fresh taste, and it is a high-quality material for roast suckling pig. In addition, most of the physiological and biochemical indicators of mini-piglets are similar to those of humans, and it is also an ideal medical experimental animal. In a previous study, the beneficial effects of tuna dark muscle hydrolysate were investigated in Holland mini-piglets [15]. Therefore, in this study, a similar pig breed was used to evaluate the beneficial effects of tuna dark muscle. The odor and volatile substances profile of pork were evaluated; the growth performance, serum lipids, and antioxidant levels were measured; and the gut microbiota was sequenced. This study provides a basis for screening safe and effective feed sources, and it promotes the healthy development of the pig farming industry.

## 2. Materials and Methods

### 2.1. Chemicals

Cooked tuna dark muscle was obtained from Ningbo Today Food Co., Ltd. (Ningbo, China). Phthalaldehyde (OPA) and 9-fluorene methyl chloroformate (FMOC) were purchased from Agilent Technologies, Inc. (Wilmington, DE, USA). The standards of amino acids and methyl butyrate solution were obtained from Sigma-Aldrich Inc. (Burlington, MA, USA). The total cholesterol (TC), total triglyceride (TG), high-density lipoprotein cholesterol (HDL-C), and low-density lipoprotein cholesterol (LDL-C) contents and the superoxide dismutase (SOD), glutathione peroxidase (GSH-Px), and malondialdehyde (MDA) indices were measured using commercial kits acquired from Nanjing Jiancheng Bioengineering Institute (Nanjing, China). Other reagents were of the needed grade and commercially available.

### 2.2. Determination of Nutritional Components of Tuna Dark Muscle

The total protein content of the tuna dark muscle was determined according to the Kjeldahl method [22]. The crude fat content of the tuna dark muscle was determined according to the Soxhlet extraction method [23]. The moisture content of the tuna dark muscle was determined according to the thermal drying method [24].

### 2.3. Analysis of Amino Acid Compositions in Dark Muscle

The tuna dark muscle (100 mg) was freeze-dried in liquid nitrogen and then crushed. The crushed samples were added to a tetrafluoroethylene digestion tube, followed by 8 mL of 6 M nitric acid. The samples were digested using closed MARS 5 microwave digestion apparatus (CEM, Matthews, NC, USA). After that, the pH was adjusted to 7.2, and the volume was diluted to 25 mL with distilled water. The amino acids composition was detected via HPLC (Agilent 1200 Series, Santa Clara, CA, USA), using pre-column derivatization with OPA (10 mg/mL, 0.5 μL for 1.0 μL sample) and FMOC (2.5 mg/mL, 0.4 μL for 1.0 μL sample).

Analysis conditions: Chromatographic column: ZORBAX Eclipse AAA column (4.6 mm × 150 mm, 3.5 μm, Agilent Technologies, CA, USA). Mobile phase A: 0.04 mol/L sodium dihydrogen phosphate (pH 7.8). Mobile phase B: acetonitrile methanol water (volume ratio 45:45:10) with the following gradient: from 0 to 1.9 min 0% B, 18.1 min 57% B, 18.8–22.3 min 100% B, 23.2 min 0% B to 28 min; the entire process lasted for 28 min. The flow rate was 2 mL/min, the column temperature was 40 °C, and the detection wavelength was 338 nm.

### 2.4. Animal Experiment

Animal experiments were conducted according to the guidelines provided by the Ningbo University Laboratory Animal Center (Ningbo, China). All protocols were approved by the Ningbo University Laboratory Animal Center under permit number NBU20190137. Twenty-four 2-month-old male Holland mini-piglets (3.1 ± 0.8 kg) were selected and fed a corn and soybean meal-based diet (Tables S1 and S2), which was purchased from Zhejiang Kaihua Hongxing Co., Ltd. (Hangzhou, China), according to the recommendation of the National Research Council (NRC, 2012) for 14 days. After 14 days of acclimatization, the pigs were randomly divided into 3 groups, with 4 replicates in each group and 2 pigs in each replicate. The pigs were fed a basal diet supplemented with 0, 2%, and 4% tuna dark muscle (control group, LD group, and HD group). During the 8-week experiment, regular and quantitative feeding (once in the morning, at midnight and in the evening, with 200 g of feed per meal) was performed. The inclusion levels of tuna dark meat were determined by previous studies with slight modifications [15,25]. The animals had free access to clean drinking water. A cage was used to weigh the pigs. After a pig was put in the cage, we waited for the pig to stop moving and calm down; then, we accurately recorded the total weight of the cage and pig. The body weights of all pigs were measured every three days.

On the last day of the experiment, the feces of each pig were collected, then immersed in liquid nitrogen immediately and stored at −80 °C. The pigs were anesthetized with an intravenous injection of sodium pentobarbital at 50 mg/kg body weight. Blood samples were collected via an inferior vena cava puncture. The serum was separated via centrifugation and stored at −80 °C. After slaughtering, longissimus dorsi, rib muscle, and tendon meat samples were rapidly excised from the right side of the carcass; immersed in liquid nitrogen; and stored at −80 °C for further analysis.

### 2.5. Identification of Odor in Pork

A sample of 0.5 g of longissimus dorsi, rib muscle, and tendon meat obtained from each group was placed in sealed bottles, and five parallel tests were conducted for each sample. Odor detection was conducted through an electronic nose (PEN3, Airsense Analytics, Schwerin, Germany). The PEN3 E-nose had ten metal oxide sensors, and the performance descriptions of the sensors are shown in Table S3. The maximum value and maximum slope of each sensor were selected as variables for further analysis. The sample injection flow rate and the carrier were 300 mL/min. The measurement time was 200 s, and the cleaning time was 300 s. The measurement data of the electronic nose were processed with the win muster data processing software that comes with PEN3 for a principal component analysis (PCA). The 298–299 s data in the stationary state were selected as the analysis point, and the horizontal and vertical coordinates included the contribution rates of PC1 and PC2.

### 2.6. Identification of Volatile Substances in Pork

A sample of 3 g of longissimus dorsi, rib muscle, and tendon meat obtained from each group was added to a 15 mL solid-phase microextraction flask, with 25 μL of 0.0898 g/mL methyl butyrate solution as the internal standard substance. An aged extraction head (65 μm polydimethylsiloxane, PDMS; Supelco, Bellefonte, PA, USA) was inserted into the sample bottle, absorbed 60 °C water for 30 min, and then inserted into the inlet. The desorption time at 220 °C was 2 min. Volatile substances were detected with Agilent 7890 gas chromatography (Agilent Technologies, California, USA) coupled to an M7-80E mass spectrometric detector (Beijing Purkinje General Instrument Co., Ltd., Beijing, China), as previously described [26].

Chromatographic conditions: DB-5 capillary column (30 m × 0.25 mm × 2.5 μm). Carrier gas: He; flow rate: 0.3 mL/min; injection: splitless mode; constant pressure: 35 kPa; inlet temperature and interface temperature: 220 °C. Programmed temperature increase: the initial column temperature was 50 °C, rose to 200 °C at 5 °C/min, held for 5 min and then rose to 250 °C at 10 °C/min, and held for 2 min; the entire process lasted for 42 min. Mass spectrometry conditions: ion source: electron impact source (EI); electron energy: 70 eV; ion source temperature: 230 °C; scanning mass range: 45–500 u.

### 2.7. Determination of Indicators Related to Blood Lipid and Antioxidant Enzyme Activity

The TC, TG, HDL-C, and LDL-C contents and the SOD, GSH-Px, and MDA indices were detected using commercial kits with an Infinite M200 Pro biochemical analyzer (Tecan Global Headquarters Tecan Group Ltd. Seestrasse, Männedorf, Switzerland).

### 2.8. Total DNA Extraction, PCR, and Sequencing in Fecal Sample

The total genomic DNA in fecal samples was extracted and quantified for amplification using a previously described method [27]. Paired-end sequencing was performed using a MiSeq system (Illumina, San Diego, CA, USA) at LC Sciences Co., Ltd. (Hangzhou, Zhejiang, China). The operational taxonomic units (OTUs) were clustered using Usearch (version 7.1). An α-diversity analysis was calculated using Mothur (version 1.36.0). A principal coordinate analysis (PCoA) was completed via Muscle (version 3.8.31). The most abundant sequences in each OTU were used for taxonomic classification using the Ribosomal Database Project (RDP) Classifier. The sequence had been deposited at the NCBI Sequence Read Archive Database under the accession number PRJNA1013740.

### 2.9. Statistical Analysis

Data are shown as the means ± standard deviation (SD). Normally distributed data (growth performance and serum indices) were assessed using an analysis of variance (*ANOVA*), followed by Tukey’s post hoc *test* (SPSS, version 19.0, Chicago, IL, USA), and data that did not meet the assumptions of the *ANOVA* (gut microbiota) were analyzed using the Mann–Whitney test (MATLAB R2012a, Natick, MA, USA). *p* < 0.05 was considered a standard criterion of statistical significance.

## 3. Results

### 3.1. Analysis of Nutritional Components of Tuna Dark Muscle

The tuna dark muscle contained about 8.31% crude fat, 18.23% crude protein, and 67.67% water. Among the essential amino acids, the content of arginine (Arg, 283.75 mg/100 g) was the highest, followed by that of threonine (Thr, 160.93 mg/100 g) and leucine (Leu, 143.53 mg/100 g) (Table 1).

### 3.2. Volatile Flavor Differences in Pork Caused by Tuna Dark Muscle

**Figure 1:**
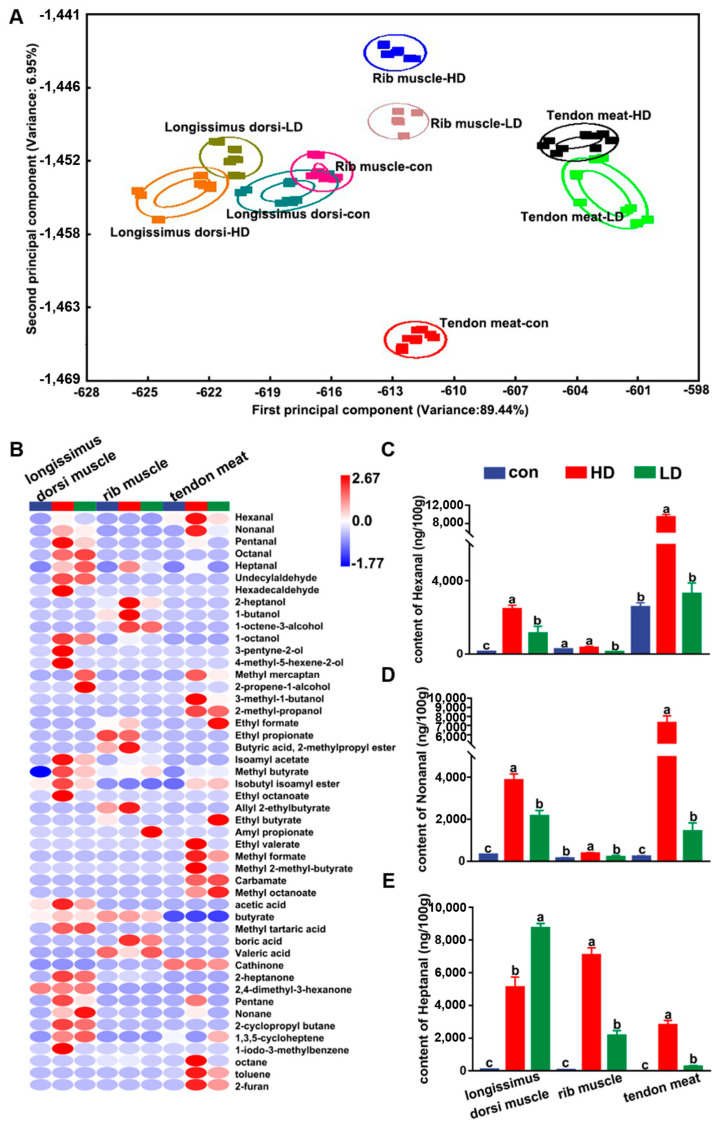
The effect of dark muscle on the odor and flavor substances of Holland mini-piglet pork. (**A**). Odor profile of meat. (**B**). Forty-eight flavor substances after dark muscle treatment in pork were obtained using GC-MS. (**C**–**E**). Distribution of three main flavor substances in each group. Different letters indicated significant differences among group at *p* < 0.05.

The variance contribution rates of the first principal component and the second principal component were 89.44% and 6.95%, respectively, and the total variance contribution rate was 96.39%. The use of tuna dark muscle at low and high dosages shifted the odor of the rib muscle and tendon meat away from that of the control group, while similar odor patterns were observed in the longissimus dorsi muscle. In addition, low and high dosages of tuna dark muscle had completely different effects on the odor of the rib muscle but partly similar effects on the odor of the longissimus dorsi muscle and tendon meat (Figure 1A).

### 3.3. Tuna Dark Muscle Increased the Content of Volatile Compounds in Pork

A total of 48 volatile flavor compounds were classified into seven different chemical classes, in which esters (15), alcohols (10), and aldehydes (7) were the predominant volatile compounds. After tuna dark muscle feeding, 27, 23, and 26 aroma compounds were released by the longissimus dorsi muscle, rib muscle, and tendon meat, respectively (Figure 1B and Table S4). Among them, hexanal, nonanal and heptanal were reported as indispensable contributors to the feeding. Compared to the control group (longissimus dorsi: 121.92 ± 2.36 ng/100 g; rib muscle: 262.16 ± 20.25 ng/100 g; and tendon meat: 2556.65 ± 235.26 ng/100 g), high-dose tuna dark muscle feeding significantly increased the content of hexanal in the longissimus dorsi (2452.27 ± 209.38 ng/100 g, *p* < 0.0001), rib muscle (342.44 ± 76.35 ng/100 g, *p =* 0.0122), and tendon meat (9420.91 ± 602.46 ng/100 g, *p* < 0.0001). The low-dose group also had increased hexanal content in the longissimus dorsi (1134.27 ± 377.32 ng/100 g, *p* < 0.0001) and tendon meat (3271.85 ± 602.67 ng/100 g, *p* = 0.0074), but the difference was not significant in the rib muscle (220.11 ± 63.25 ng/100 g, *p* = 0.0950). Compared to the control group, the tuna dark muscle treatment significantly increased the contents of nonanal and heptanal in the longissimus dorsi (LD: 2140.37 ± 268.48 ng/100 g, *p* < 0.0001 and 8730.18 ± 275.48 ng/100 g, *p* < 0.0001; HD: 3846.48 ± 299.38 ng/100 g, *p* < 0.0001 and 5108.13 ± 627.98 ng/100 g, *p* < 0.0001), rib muscle (LD: 201.57 ± 71.24 ng/100 g, *p* = 0.0326 and 2159.29 ± 302.41 ng/100 g, *p* < 0.0001; HD: 369.34 ± 37.24 ng/100 g, *p* < 0.0001 and 7072.87 ± 444.25 ng/100 g, *p* < 0.0001), and tendon meat (LD: 1421.56 ± 405.25 ng/100 g, *p* < 0.0001 and 265.53 ± 64.26 ng/100 g, *p* < 0.0001; HD: 7270.85 ± 803.21 ng/100 g, *p* < 0.0001 and 2812.64 ± 267.26 ng/100 g, *p* < 0.0001) (Figure 1C–E).

### 3.4. Tuna Dark Muscle Significantly Increased the Weight of Holland Mini-Piglets

Compared to the control group, the low and high dosages of tuna dark muscle feeding significantly increased the average daily weight gain from 0.13 ± 0.02 kg/d in the control group to 0.19 ± 0.01 kg/d (*p* < 0.0001) and 0.16 ± 0.003 kg/d (*p =* 0.0009) in the HD and LD groups, respectively (Table 2). In addition, the intake of dark muscle showed no significant effect on the organ indices of liver (*p* = 0.7385), kidney (*p* = 0.3034), spleen (*p* = 0.3985), and heart (*p* = 0.1228) (Table 2).

### 3.5. Tuna Dark Muscle Enhanced the Antioxidant Capacity of Holland Mini-Piglets

Compared to the control group, the tuna dark muscle treatment did not significantly affect the levels of TG (*p* = 0.6670), TC (*p* = 0.0980), HDL (*p* = 0.3799), and LDL (*p* = 0.7578) in serum (Table 3), while the dark muscle treatment significantly increased the antioxidant capacity of the Holland mini-piglets (*p* < 0.0001 for SOD and GSH-Px activities and *p* = 0.0001 for MDA content) (Table 3). Compared with the control group, the HD group had significantly increased SOD (27.35 ± 2.09 U/mL, *p* = 0.0015) and GSH-Px activities (701.59 ± 59.8 U/mL, *p* = 0.0016) and decreased MDA content (1.46 ± 0.25 nmol/mL, *p* = 0.00019). The LD group had significantly decreased GSH-Px activity (433.64 ± 10.02 U/mL, *p* = 0.0165) and MDA content (1.51 ± 0.27 nmol/mL, *p* = 0.0006). Compared with the control group, SOD activity (22.84 ± 1.38 U/mL, *p* = 0.0511) decreased in the LD group, but the difference was not significant.

### 3.6. Tuna Dark Muscle Modulated the Gut Microbiota in Holland Mini-Piglets

**Figure 2:**
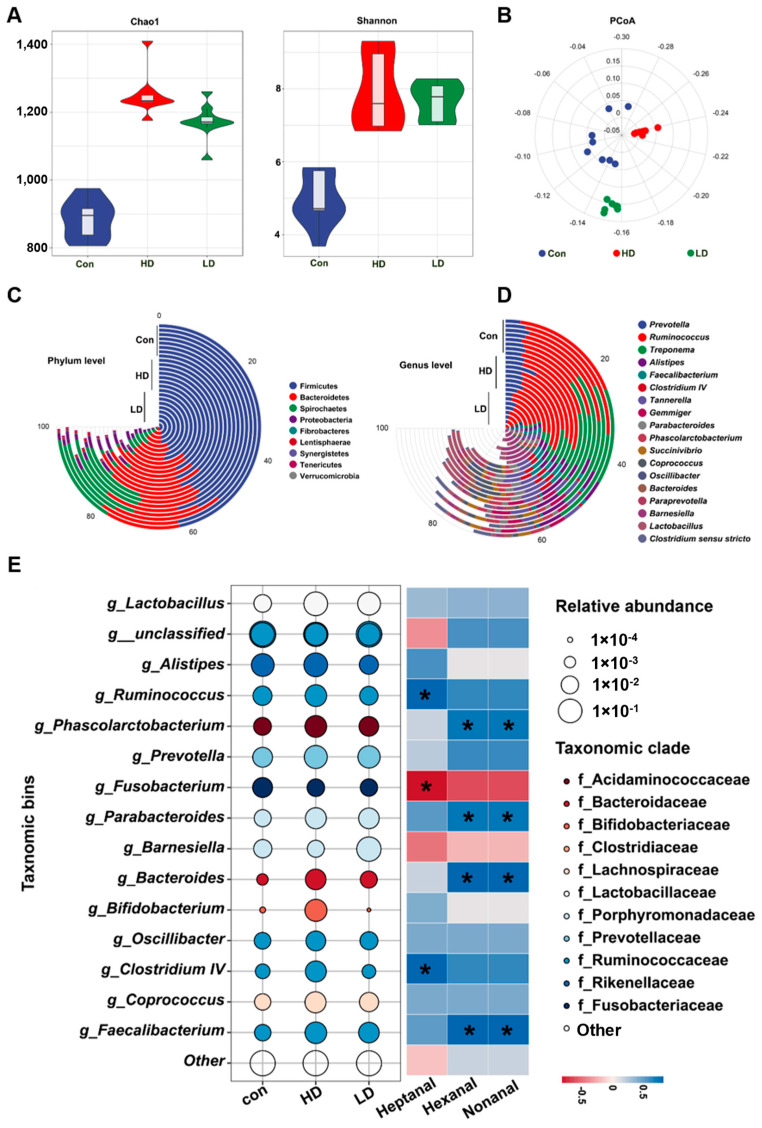
The effect of dark muscle on gut microbiota. (**A**). The alpha diversity of the gut microbiota in three groups. (**B**). Variations in the gut microbiota structures of pigs treated with dark muscle via weighted UniFrac PCoA. (**C**). RDP classifications of microbial composition at the phylum level and genus level. (**D**). After tuna dark muscle treatment, sixteen genera with high abundance in the gut microbiota were obtained. The larger the circle in the bubble chart is, the higher the abundance. (**E**). Spearman’s correlation analysis of 16 genera with hexanal and nonanal. Red indicates a negative correlation, and blue indicates a positive correlation. * *p* < 0.05.

Changes in gut microbiota richness (Chao1) and diversity (Shannon index) were analyzed to elucidate the contribution of the tuna dark muscle to the Holland mini-piglets. Compared to the control group, both the HD and LD groups had increased richness (LD: 1172.58 ± 50.27, *p* < 0.0001; HD: 1250.48 ± 60.52, *p* < 0.0001) and diversity (LD: 7.67 ± 0.50, *p* < 0.0001; HD: 7.92 ± 1.01, *p* < 0.0001) of the gut microbiota, while the HD group showed a relatively higher richness and lower diversity than the LD group (Figure 2A). In addition, the PCoA analysis indicated that the tuna dark muscle treatment altered the structure of the gut microbiota, with both the low and high dosages showing similar but not identical modulation effects on the gut microbiota (Figure 2B).

As presented in Figure 2C, the phylum-level analysis showed that the most abundant phyla in the control group included *Firmicutes*, *Bacteroidetes*, *Spirochaetes*, and *Proteobacteria*. There were no significant differences in the abundance of *Firmicutes* in the three groups. However, compared with control group, the HD and LD groups showed a significant increase in the relative abundances of *Bacteroidetes* (*p* < 0.0001 and *p* = 0.0013 in the HD and LD groups) and *Proteobacteria* (*p* = 0.0005 and *p* = 0.00044) and a significant reduction in the relative abundance of *Spirochaetes* (*p* < 0.0001 and *p* < 0.0001) (Figure 2C and Table S5).

At the genus level, compared to the control group, the HD group showed significantly increased abundances of *Prevotella* (*p* = 0.00011), *Ruminococcus* (*p* = 0.00014), *Faecalibacterium* (*p* < 0.0001), *Clostridium IV* (*p* < 0.0001), *Parabacteroides* (*p* < 0.0001), *Coprococcus* (*p* < 0.0001), *Oscillibacter* (*p* < 0.0001), *Paraprevotella* (*p* < 0.0001), and *Lactobacillus* (*p* < 0.0001) and reduced abundances of *Treponema* (*p* < 0.0001) and *Clostridium sensu stricto* (*p* = 0.0002). In addition, the abundances of *Faecalibacterium* (*p* < 0.0001), *Parabacteroides* (*p* < 0.0001), *Lactobacillus* (*p* < 0.0001), and *Oscillibacter* (*p* < 0.0001) were significantly increased in the LD group, while the abundances of *Treponema* (*p* < 0.0001) and *Alistipes* (*p* < 0.0001) were significantly decreased in the LD group (Figure 2D and Table S6).

### 3.7. Key Genus Responding to Tuna Dark Muscle Feeding in Holland Mini-Piglets

A total of 16 key genera responding to the tuna dark muscle feeding were identified via a redundancy analysis (RDA). In total, 13 and 10 genera were increased in the HD and LD groups, respectively, compared with the control group, while 2 and 5 genera were decreased (Figure 2D and Table S7).

Spearman’s correlation analysis was performed to correlate these 16 genera with the main flavor substances. Seven key genera were significantly correlated with at least one volatile substance (Figure 2E). *Ruminococcus*, *Phascolarctobacterium*, *Parabacteroides*, *Bacteroides*, *Clostridium IV*, and *Faecalibacterium* were positively correlated with the main volatile substances, and their relative abundances were higher in the HD group. *Fusobacterium* was negatively correlated with the main volatile substances, and its relative abundance was lower in the HD and LD groups than in the control group.

## 4. Discussion

Many studies have reported that marine protein can be used as a nutritional supplement to improve swine growth performance, but the mechanism has not been clarified [16,28,29]. Amino acids are not only precursors for protein synthesis but also guarantee the intestinal health, function, and growth rate of pigs [30,31]. Studies have shown that the dietary supplementation of glutamate, glycine, and arginine reduces cellular oxidative stress [32]. Arginine (Arg) is a conditional essential amino acid, and it can protect the body from oxidative stress and inflammation, activate the mTOR signaling pathway in intestinal tissue, and regulate intestinal inflammation [33]. In a previous study, arginine feeding improved the production performance of pigs during pregnancy, lactation, weaning, and growth periods [34]. Furthermore, the addition of leucine to diets increased the synthesis of tissue protein in weanling pigs [35]. Threonine is critical for maintaining gut immunological functions [36]. In this study, tuna dark muscle was found to be rich in arginine, threonine and leucine (Table 1), and the addition of tuna dark muscle increased the supply of these amino acid, which contributed to the growth performance of the pigs.

It is well known that enhancing antioxidative status could improve meat quality. Therefore, dietary natural antioxidants, such as chlorogenic acid, α-tocopheryl acetate, and green tea catechins, decrease lipid peroxidation and improve antioxidative status, along with improving meat quality [13,37]. Tuna dark muscle hydrolysate showed significant antioxidant activity, as previously described [10], and, in this study, the direct dietary supplementation of tuna dark muscle at a high dosage increased SOD and GSH-Px levels and decreased MDA levels (Table 3), which might contribute to improved pork quality.

Different pig breeds show different volatile compounds [38]. Moreover, the volatile substances profile of pork can easily be manipulated through feed, and the accumulation of plant-derived compounds from feed has shown an influence on the volatile composition of pork [39]. Therefore, in this study, an 8-week diet of tuna dark muscle led to a volatile substances profile different to that of the control group (Figure 1A). Furthermore, hexanal, nonanal, and heptanal, which show grass, putty, and fatty notes, come from lipid oxidation and may contribute to the oil aroma of pork [40], and their abundances significantly increased in response to the tuna dark muscle feeding (Figure 1C–E).

The gut microbiota plays an important role in the digestion and absorption of nutrients, as well as in intestinal health. Alfalfa silage feeding was found to modulate the gut microbiota, along with improving meat quality [4]. High-energy feeding affected the meat quality of Tibetan pigs, and this was closely associated with alteration in the gut microbiota [41]. In addition, several specific strains associated with feeding behavior at different stages of swine were identified [42]. In this study, 16S rDNA sequencing indicated different overall structures of the gut microbiota among the control and tuna dark muscle-treated groups. *Prevotella* exists in the intestinal tract, helping to decompose protein and carbohydrates, as well as helping the body absorb energy [43]. *Ruminococcus*, *Faecalibacterium*, *Coprococcus*, *Oscillibacter*, and *Lactobacillus* are butyric-acid-producing bacterium. Butyric acid can provide energy to the body through fatty acid oxidation and is the main energy source for intestinal epithelial cells. Butyric acid is closely related to body health and has significant implications for regulating intestinal health and inhibiting inflammation. Butyrate is often added in the breeding industry to protect the healthy growth of animals, such as preventing diarrhea in weaned piglets, regulating the gut microbiota of chickens and enhancing their immunity [44]. In addition, *Lactobacillus* stabilize the intestinal microecological balance but also use their own SOD enzymes to scavenge free radicals and effectively inhibit the occurrence of oxidative stress in the gut [45]. Previous studies have shown that long-term supplementation with protein-rich foods leads to an increase in the abundance of *Bacteroidetes* [46]. *Fusobacterium* produces lipopolysaccharide, endotoxin, and hemolysin. Therefore, it is considered a conditional pathogen with strong toxicity, and it can cause various infectious diseases [47]. In a previous study, *Clostridium sensu stricto* was significantly increased in DSS-induced colitis mice, indicating that *Clostridium sensu stricto* has pro-inflammatory effects [48]. In this study, the tuna dark muscle treatment significantly increased the abundances of *Prevotella*, *Ruminococcus*, *Faecalibacterium*, *Coprococcus*, *Oscillibacter*, *Lactobacillus*, and *Bacteroidetes* and decreased the abundances of *Fusobacterium* and *Clostridium sensu stricto*. These key bacteria play important roles in improving pig growth performance, enhancing antioxidant levels, and regulating intestinal homeostasis.

This study indicates that supplementing mini-piglets with tuna dark muscle has beneficial effects on their growth performance and the quality of pork. In addition, tuna dark muscle supplementation can modulate the gut microbiota. However, there are some limitations to this study. Firstly, the mini-pig used in this study is not a common meat producer at a large scale, and, secondly, the meat quality, including the composition of fatty acids, proteins, and amino acids, should be systematically evaluated. Therefore, further studies should focus on meat producers and quantitatively evaluate pork quality in response to dietary tuna dark muscle supplementation.

## 5. Conclusions

In this study, both 2% and 4% cooked tuna dark muscle supplementation shifted the odor of rib muscle and tendon meat away from that of the control group and increased the hexanal, nonanal, and heptanal contents, which lead to improved pork quality. In addition, dietary tuna dark muscle modulated the gut microbiota, with an increased abundance of beneficial bacteria and a reduced abundance of harmful bacteria. This feeding strategy not only reduces production costs of pork but also utilizes tuna processing by-products in an environmentally friendly way. However, this feeding strategy has some challenges. Firstly, it is more suitable for areas with a tuna processing industry, which will produce a large amount of tuna processing by-products. Secondly, forage palatability and the underlying mechanism remain unclear, which will be taken into consideration in future studies.

## Figures and Tables

**Table 1:** Amino acid composition of tuna dark muscle.

	Amino Acid	Content (mg/100 g)
Essential amino acids	Arginine (Arg)	283.75 ± 3.47
	Threonine (Thr)	160.93 ± 3.68
	Leucine (Leu)	143.53 ± 2.48
	Lysine (Lys)	108.3 ± 5.68
	Isoleucine (Ile)	90.84 ± 2.45
	Histidine (His)	73.66 ± 5.24
	Valine (Val)	62.8 ± 2.67
	Phenylalanine (Phe)	18.99 ± 1.07
	Methionine (Met)	10.03 ± 0.68
	Tryptophan (Trp)	-
Non-essential amino acids	Glycine (Gly)	128.8 ± 4.47
	Glutamic acid (Glu)	121.71 ± 3.46
	Proline (Pro)	119.88 ± 4.76
	Serine (Ser)	105.79 ± 5.58
	Alanine (Ala)	102.5 ± 3.97
	Aspartic acid (Asp)	97.23 ± 1.43
	Tyrosine (Tyr)	87.47 ± 5.36
	Asparagine (Asn)	73.35 ± 3.58
	Cystine (Cys)	32.14 ± 3.25
	Glutarnine (Gln)	-

**Table 2:** The effect of dark muscle on daily body weight gain and organ indices. Data are presented as means ± SD; different letters indicated significant differences among group at *p* < 0.05.

	Control	HD	LD	*p*-Value
Daily body weight gain (kg/d)	0.13 ± 0.02 a	0.19 ± 0.01 b	0.16 ± 0.003 c	<0.0001
Liver index (g/kg)	27.67 ± 2.08 a	28.01 ± 1.01 a	28.23 ± 0.93 a	0.7385
Kidney index (g/kg)	6.73 ± 0.51 a	6.43 ± 0.71 a	6.3 ± 0.4 a	0.3034
Spleen index (g/kg)	1.93 ± 0.71 a	2.57 ± 1.4 a	2.13 ± 0.46 a	0.3985
Heart index (g/kg)	4.9 ± 0.36 a	5.07 ± 0.23 ab	5.33 ± 0.55 b	0.1228

**Table 3:** The effect of dark muscle on blood lipid indicators and antioxidant enzyme activity. Data are presented as means ± SD; different letters indicated significant differences among group at *p* < 0.05.

	Control	HD	LD	*p*-Value
TG (mmol/L)	0.51 ± 0.17 a	0.49 ± 0.28 a	0.42 ± 0.15 a	0.6670
TC (mmol/L)	2.67 ± 0.78 a	3.29 ± 0.28 b	3.04 ± 0.46 ab	0.0980
HDL (mmol/L)	12.45 ± 1.19 a	11.21 ± 1.26 a	12.83 ± 3.74 a	0.3799
LDL (mmol/L)	1.16 ± 0.43 a	1.22 ± 0.21 a	1.28 ± 0.28 a	0.7578
SOD (U/mL)	24.13 ± 1.01 a	27.35 ± 2.09 b	22.84 ± 1.38 a	<0.0001
GSH-Px (U/mL)	535.23 ± 105.02 a	701.59 ± 59.8 b	433.64 ± 10.02 c	<0.0001
MDA (nmol/mL)	2.05 ± 0.22 a	1.46 ± 0.25 b	1.51 ± 0.27 b	0.0001

## Data Availability

The original contributions presented in the study are included in the article/supplementary material, further inquiries can be directed to the corresponding authors.

## Supplementary Materials

The following supporting information can be downloaded at: https://www.mdpi.com/article/10.3390/foods13101577/s1.

## References

[1] Wang L., Zhang S., Huang Y., You W., Zhou Y., Chen W., Sun Y., Yi W., Sun H., Xie J. (2022). CLA improves the lipo-nutritional quality of pork and regulates the gut microbiota in Heigai pigs. Food Funct..

[2] Pereira P.M., Vicente A.F. (2013). Meat nutritional composition and nutritive role in the human diet. Meat Sci..

[3] Sampath V., Park J.H., Kim I.H. (2023). Synbiotic-Glyconutrient Additive Reveals a Conducive Effect on Growth Performance, Fatty Acid Profile, Sensory Characteristics, and Texture Profile Analysis in Finishing Pig. Foods.

[4] Xu J., Liu X., Geng H., Liu R., Li F., Ma J., Liu M., Liu B., Sun H., Ma S. (2023). Alfalfa Silage Diet Improves Meat Quality by Remodeling the Intestinal Microbes of Fattening Pigs. Foods.

[5] Kouba M., Enser M., Whittington F.M., Nute G.R., Wood J.D. (2003). Effect of a high-linolenic acid diet on lipogenic enzyme activities, fatty acid composition, and meat quality in the growing pig. J. Anim. Sci..

[6] Costa M., Cardoso C., Afonso C., Bandarra N.M., Prates J.A.M. (2021). Current knowledge and future perspectives of the use of seaweeds for livestock production and meat quality: A systematic review. J. Anim. Physiol. Anim. Nutr..

[7] Nowacka-Woszuk J. (2020). Nutrigenomics in livestock-recent advances. J. Appl. Genet..

[8] Chamorro F., Cassani L., Garcia-Oliveira P., Barral-Martinez M., Jorge A.O.S., Pereira A.G., Otero P., Fraga-Corral M., Oliveira M.B.P.P., Prieto M.A. (2024). Health benefits of bluefin tuna consumption: (*Thunnus thynnus*) as a case study. Front. Nutr..

[9] Maeda H., Hosomi R., Fukuda M., Ikeda Y., Yoshida M., Fukunaga K. (2017). Dietary Tuna Dark Muscle Protein Attenuates Hepatic Steatosis and Increases Serum High-Density Lipoprotein Cholesterol in Obese Type-2 Diabetic/Obese KK-A(y) Mice. J. Food Sci..

[10] Han J., Tang S., Li Y., Bao W., Wan H., Lu C., Zhou J., Cheong L., Su X. (2018). In silico analysis and in vivo tests of the tuna dark muscle hydrolysate anti-oxidation effect. RSC Adv..

[11] Chi C.F., Hu F.Y., Wang B., Li Z.R., Luo H.Y. (2015). Influence of Amino Acid Compositions and Peptide Profiles on Antioxidant Capacities of Two Protein Hydrolysates from Skipjack Tuna (*Katsuwonus pelamis*) Dark Muscle. Mar. Drugs.

[12] Masuda J., Umemura C., Yokozawa M., Yamauchi K., Seko T., Yamashita M., Yamashita Y. (2018). Dietary Supplementation of Selenoneine-Containing Tuna Dark Muscle Extract Effectively Reduces Pathology of Experimental Colorectal Cancers in Mice. Nutrients.

[13] Wang W., Wen C., Guo Q., Li J., He S., Yin Y. (2021). Dietary Supplementation with Chlorogenic Acid Derived from *Lonicera macranthoides* Hand-Mazz Improves Meat Quality and Muscle Fiber Characteristics of Finishing Pigs via Enhancement of Antioxidant Capacity. Front. Physiol..

[14] Zhang C., Luo J., Yu B., Zheng P., Huang Z., Mao X., He J., Yu J., Chen J., Chen D. (2015). Dietary resveratrol supplementation improves meat quality of finishing pigs through changing muscle fiber characteristics and antioxidative status. Meat Sci..

[15] Zhou J., Yang M., Han J., Lu C., Li Y., Su X. (2019). Effects of dietary tuna dark muscle enzymatic hydrolysis and cooking drip supplementations on growth performance, antioxidant activity and gut microbiota modulation of Bama mini-piglets. RSC Adv..

[16] Howe P.R., Downing J.A., Grenyer B.F., Grigonis-Deane E.M., Bryden W.L. (2002). Tuna fishmeal as a source of DHA for n-3 PUFA enrichment of pork, chicken, and eggs. Lipids.

[17] Castellano C.A., Audet I., Laforest J.P., Matte J.J., Suh M. (2011). Fish oil diets alter the phospholipid balance, fatty acid composition, and steroid hormone concentrations in testes of adult pigs. Theriogenology.

[18] Han J., Huang Z., Tang S., Lu C., Wan H., Zhou J., Li Y., Ming T., Jim Wang Z., Su X. (2020). The novel peptides ICRD and LCGEC screened from tuna roe show antioxidative activity via Keap1/Nrf2-ARE pathway regulation and gut microbiota modulation. Food Chem..

[19] Han J., Wang X., Tang S., Lu C., Wan H., Zhou J., Li Y., Ming T., Wang Z.J., Su X. (2020). Protective effects of tuna meat oligopeptides (TMOP) supplementation on hyperuricemia and associated renal inflammation mediated by gut microbiota. FASEB J. Off. Publ. Fed. Am. Soc. Exp. Biol..

[20] Wang X., Tsai T., Deng F., Wei X., Chai J., Knapp J., Apple J., Maxwell C.V., Lee J.A., Li Y. (2019). Longitudinal investigation of the swine gut microbiome from birth to market reveals stage and growth performance associated bacteria. Microbiome.

[21] Chen C., Zhou Y., Fu H., Xiong X., Fang S., Jiang H., Wu J., Yang H., Gao J., Huang L. (2021). Expanded catalog of microbial genes and metagenome-assembled genomes from the pig gut microbiome. Nat. Commun..

[22] Saxton R., McDougal O.M. (2021). Whey Protein Powder Analysis by Mid-Infrared Spectroscopy. Foods.

[23] Nahar L., Uddin S.J., Alam M.A., Sarker S.D. (2021). Extraction of naturally occurring cannabinoids: An update. Phytochem. Anal. PCA.

[24] Alibas I., Yilmaz A. (2022). Microwave and convective drying kinetics and thermal properties of orange slices and effect of drying on some phytochemical parameters. J. Therm. Anal. Calorim..

[25] Grabež V., Devle H., Kidane A., Mydland L.T., Øverland M., Ottestad S., Berg P., Kåsin K., Ruud L., Karlsen V. (2023). Sugar Kelp (*Saccharina latissima*) Seaweed Added to a Growing-Finishing Lamb Diet Has a Positive Effect on Quality Traits and on Mineral Content of Meat. Foods.

[26] Chen G., Cai Y., Su Y., Wang D., Pan X., Zhi X. (2021). Study of meat quality and flavour in different cuts of Duroc-Bamei binary hybrid pigs. Vet. Med. Sci..

[27] Huang Y., Fan S., Lu G., Sun N., Wang R., Lu C., Han J., Zhou J., Li Y., Ming T. (2021). Systematic investigation of the amino acid profiles that are correlated with xanthine oxidase inhibitory activity: Effects, mechanism and applications in protein source screening. Free Radic. Biol. Med..

[28] Thuy N.T., Ha N.C. (2016). Effect of replacing marine fish meal with catfish (*Pangasius hypophthalmus*) by-product protein hydrolyzate on the growth performance and diarrhoea incidence in weaned piglets. Trop. Anim. Health Prod..

[29] Altmann B.A., Neumann C., Rothstein S., Liebert F., Mörlein D. (2019). Do dietary soy alternatives lead to pork quality improvements or drawbacks? A look into micro-alga and insect protein in swine diets. Meat Sci..

[30] Millet S., Langendries K., Aluwé M., De Brabander D.L. (2011). Effect of amino acid level in the pig diet during growing and early finishing on growth response during the late finishing phase of lean meat type gilts. J. Sci. Food Agric..

[31] Mou Q., Yang H.S., Yin Y.L., Huang P.F. (2019). Amino Acids Influencing Intestinal Development and Health of the Piglets. Animals.

[32] Duan J., Yin J., Ren W., Liu T., Cui Z., Huang X., Wu L., Kim S.W., Liu G., Wu X. (2016). Dietary supplementation with L-glutamate and L-aspartate alleviates oxidative stress in weaned piglets challenged with hydrogen peroxide. Amino Acids.

[33] Wu G. (2014). Dietary requirements of synthesizable amino acids by animals: A paradigm shift in protein nutrition. J. Anim. Sci. Biotechnol..

[34] Che D., Adams S., Zhao B., Qin G., Jiang H. (2019). Effects of Dietary L-arginine Supplementation from Conception to Post-Weaning in Piglets. Curr. Protein Pept. Sci..

[35] Yin Y., Yao K., Liu Z., Gong M., Ruan Z., Deng D., Tan B., Wu G. (2010). Supplementing L-leucine to a low-protein diet increases tissue protein synthesis in weanling pigs. Amino Acids.

[36] Yang Z., Liao S.F. (2019). Physiological Effects of Dietary Amino Acids on Gut Health and Functions of Swine. Front. Vet. Sci..

[37] Mason L.M., Hogan S.A., Lynch A., O’Sullivan K., Lawlor P.G., Kerry J.P. (2005). Effects of restricted feeding and antioxidant supplementation on pig performance and quality characteristics of longissimus dorsi muscle from Landrace and Duroc pigs. Meat Sci..

[38] Han D., Zhang C.H., Fauconnier M.L., Mi S. (2020). Characterization and differentiation of boiled pork from Tibetan, Sanmenxia and Duroc × (Landrac × Yorkshire) pigs by volatiles profiling and chemometrics analysis. Food Res. Int..

[39] Bleicher J., Ebner E.E., Bak K.H. (2022). Formation and Analysis of Volatile and Odor Compounds in Meat-A Review. Molecules.

[40] Petričević S., Marušić Radovčić N., Lukić K., Listeš E., Medić H. (2018). Differentiation of dry-cured hams from different processing methods by means of volatile compounds, physico-chemical and sensory analysis. Meat Sci..

[41] Zhu Y., Cidan Y., Sun G., Luo C., Duan J., Shi B., Ma T., Tang S., Zhong R., Chen L. (2022). Different feeding patterns affect meat quality of Tibetan pigs associated with intestinal microbiota alterations. Front. Microbiol..

[42] He Y., Tiezzi F., Howard J., Huang Y., Gray K., Maltecca C. (2022). Exploring the role of gut microbiota in host feeding behavior among breeds in swine. BMC Microbiol..

[43] Ley R.E. (2016). Gut microbiota in 2015: Prevotella in the gut: Choose carefully. Nat. Rev. Gastroenterol. Hepatol..

[44] Mallo J.J., Balfagón A., Gracia M.I., Honrubia P., Puyalto M. (2012). Evaluation of different protections of butyric acid aiming for release in the last part of the gastrointestinal tract of piglets. J. Anim. Sci..

[45] Wang H., Shi P., Huang C., Liu Q. (2016). Maresin 1 ameliorates iron-deficient anemia in IL-10(-/-) mice with spontaneous colitis by the inhibition of hepcidin expression though the IL-6/STAT3 pathway. Am. J. Transl. Res..

[46] Wu G.D., Chen J., Hoffmann C., Bittinger K., Chen Y.Y., Keilbaugh S.A., Bewtra M., Knights D., Walters W.A., Knight R. (2011). Linking long-term dietary patterns with gut microbial enterotypes. Science.

[47] Bi D., Zhu Y., Gao Y., Li H., Zhu X., Wei R., Xie R., Cai C., Wei Q., Qin H. (2022). Profiling Fusobacterium infection at high taxonomic resolution reveals lineage-specific correlations in colorectal cancer. Nat. Commun..

[48] Li M., Li Q., Abdlla R., Chen J., Yue X., Quek S.Y. (2023). Donkey whey proteins ameliorate dextran sulfate sodium-induced ulcerative colitis in mice by downregulating the S100A8-TRAF6-NF-κB axis-mediated inflammatory response. Food Sci. Hum. Wellness.

