# Supplementary material for: Tuna Dark Muscle Feeding Improved the Meat Quality of Holland Mini-Piglets and Modulated the Gut Microbiota
Source: Foods. 2024 May 18;13(10):1577. doi: 10.3390/foods13101577 (PMC11121099; doi:10.3390/foods13101577)
Supplement: Supplementary file 1 [file foods-13-01577-s001.zip › foods-2994450-supplementary.pdf]

Supporting information for

Tuna dark muscle feeding improved the meat quality of Holland mini-piglets and modulated the gut microbiota

Chenyang Lu[1,2]*, Yuanming Zhang[2], Yang Qin[2], Jun Zhou[2], Yanbo Wang[1], Xiurong Su[2], Jiaojiao Han[2]*

[1] School of Food Science and Biotechnology, Zhejiang Gongshang University, Hangzhou 310018, China.

[2] School of Marine Science, Ningbo University, Ningbo 315832, China

* Corresponding author

Dr. Chenyang Lu

School of Food Science and Biotechnology, Zhejiang Gongshang University, 18 Xuezheng Road, Hangzhou 310018, China

Dr. Jiaojiao Han

School of Marine Science, Ningbo University, 169 Qixing South Road, Ningbo 315832, China

Tel/Fax: +86 574 87608368

E-mail address: hanjiaojiao@nbu.edu.cn

Table S1. The ingredients levels of chow diet used in this study.

**Ingredients**	**Content (%)**
Corn	51.7
Soybean meal	21.2
Wheat	15
Soybean oil	0.9
Fish meal	6
Minerals mixture [a]	5.15
Vitamins mixture [b]	0.05
Total	100

a Iron=84 mg/kg, zinc=103 mg/kg, copper=32 mg/kg, manganese=34 mg/kg, selenium=0.19 mg/kg, iodine=0.7 mg/kg.

b Vitamin A=8000 IU/kg, vitamin B1=9.7 mg/kg, vitamin B2=2.6 mg/kg, vitamin B6=10.4 mg/kg, vitamin B12=0.09 mg/kg, vitamin D3=121 IU/kg, vitamin E=16 IU/kg, vitamin K3=2.9 mg/kg, nicotinic acid=15.2 mg/kg, folic acid=0.66 mg/kg, biotin=0.32 mg/kg.

Table S2. The nutrient levels of chow diet used in this study.

Nutrients	Content (%)
Dry matter	83.86
Crude protein	19.6
Crude fat	3.64
Crude fiber	3.01
Ash	3.00
Neutral detergent fiber	9.58
Acid detergent fiber	4.22
Starch	40.83
Calcium	0.34
Phosphorus	0.53
Available phosphorus	0.29
Lysine	1.28
Methionine	0.39
Threonine	0.82

Table S3. Performance descriptions of sensors in PEN3.

Number	Sensor name	Object substances
S1	W1C	Aromatics
S2	W5S	Nitrogen oxides
S3	W3C	Ammonia and aromatics
S4	W6S	Hydrogen
S5	W5C	Aromatics and aliphatics
S6	W1S	Methane
S7	W1W	Sulfides
S8	W2S	Alcohols, aldehydes and ketones
S9	W2W	Aromatics and organic sulfides
S10	W3S	Aliphatics

Table S4. The content of each volatile component after dark muscle treatment.

Compound	longissimus dorsi muscle(ng/100g)			rib muscle（ng/100g）			tendon meat（ng/100g）		
	con	HD	LD	con	HD	LD	con	HD	LD
Hexanal	121.92±2.36	2452.27±209.38	1134.27±377.32	262.16±20.25	342.44±76.35	220.11±63.25	2556.65±235.26	9420.91±602.46	3271.85±602.67
Nonanal	309.87±2.39	3846.48±299.38	2140.37±268.48	134.93±35.26	369.34±37.24	201.57±71.24	224.31±37.26	7270.85±803.21	1421.56±405.25
Pentanal	53.73±1.57	6200.19±537.96	2177.48±258.28	—	—	—	—	1375.41±302.36	92.53±20.36
Octanal	—	5277.33±469.38	6310.68±375.59	—	—	—	—	—	—
Heptanal	87.91±1.47	5108.13±627.98	8730.18±275.48	66.26±6.25	7072.87±444.25	2159.29±302.41	—	2812.64±267.26	265.53±64.26
Undecylaldehyde	—	6757.17±682.37	6143.62±537.63	—	—	—	—	—	—
Hexadecaldehyde	34.38±1.36	5877.37±839.54	—	—	—	—	—	—	—
2-heptanol	—	—	—	122.57±178.36	1287.26±58.34	340.82±13.25	—	—	—
1-butanol	—	—	—	327.19±56.03	1288.85±66.34	23.61±2.47	—	—	—
1-octene-3-alcohol	—	—	—	23.84±2.39	276.64±76.79	233.44±23.46	—	—	—
1-octanol	92±1.97	1044.59±173.58	787.46±48.27	—	211.84±66.24	121.28±32.69	—	—	—
3-pentyne-2-ol	—	3639.47±179.38	—	—	—	—	—	—	—
4-methyl-5-hexene-2-ol	—	9277.67±183.47	—	—	—	—	—	—	—
Methyl mercaptan	32.77±0.96	—	3675.87±264.99	—	—	—	30.92±10.25	3715.24±903.37	1269.14±302.67
2-propene-1-alcohol	—	—	4655.53±165.38	—	—	—	—	—	—
3-methyl-1-butanol	—	—	—	—	—	—	75.62±13.26	9551.22±1001.36	1557.44±699.36
2-methyl-propanol	—	—	—	—	—	—	46.43±8.25	1984.21±340.77	1715.26±490.02

Ethyl formate	—	—	—	262.33±67.47	500.24±68.24	34.15±11.24	—	53.74±20.36	1347.53±306.67
Ethyl propionate	—	—	—	1635.13±286.26	1426.44±57.03	—	—	—	—
Butyric acid, 2-methylpropyl ester	—	—	—	172.18±53.72	368.24±40.24	27.44±13.24	—	—	—
Isoamyl acetate	45.37±1.86	1588.93±158.89	677.55±279.27	—	255.74±50.46	211.79±56.35	—	—	—
Methyl butyrate	22450±74.38	22450.58±99.57	22450.38±75.93	22450.26±60.27	22450.29±75.01	22450.35±73.25	22450.15±80.24	22450.26±59.36	22450.26±97.35
Isobutyl isoamyl ester	1980.79±97.37	4750.87±98.26	2359.93±389.28	420.93±56.28	200.81±68.23	29.46±14.25	—	2454.34±602.68	2769.94±690.29
Ethyl octanoate	—	5058.77±95.37	—	—	—	—	—	—	—
Allyl 2-ethylbutyrate	—	—	—	222.69±52.26	421.97±53.56	25.34±10.35	—	—	—
Ethyl butyrate	—	—	—	615.26±34.21	—	—	—	121.54±36.97	2679.64±683.68
Amyl propionate	—	—	—	—	—	234.72±61.45	—	—	—
Ethyl valerate	—	—	—	—	—	—	—	33.73±11.03	—
Methyl formate	—	—	—	—	—	—	—	2763.56±893.56	1527.54±502.89
Methyl 2-methyl-butyrate	—	—	—	—	—	—	—	45.74±28.35	—
Carbamate	—	—	—	—	—	—	41.55±23.44	2151.24±246.7	2363.74±957.57
Methyl octanoate	—	—	—	—	—	—	34.64±2.34	1820.57±305.36	3070.65±779.36
acetic acid	1980.76±84.86	6318.94±78.36	3280.27±552.71	—	—	—	—	—	—
butyrate	5302±102.57	6607.49±111.36	5817.93±324.48	7935.26±509.26	7708.46±39.26	6535.24±350.23	1507.33±302.15	1324.26±793.95	1203.24±305.26
Methyl tartaric acid	46.14±1.58	9289.37±156.78	9959.32±276.27	—	—	—	—	—	—
boric acid	—	—	—	—	182.44±49.25	126.43±73.26	—	—	—
Valeric acid	—	—	—	2152.25±602.25	1009.75±48.68	2328.26±267.36	—	—	—
Cathinone	—	—	—	180.45±35.22	205.19±61.03	222.36±36.27	2097.69±208.37	2035.45±308.63	1802.46±496.27
2-heptanone	71.64±2.37	2272.85±101.26	1870.94±247.27	198.59±73.26	296.51±30.35	216.26±73.72	—	—	—
2,4-dimethyl-3-hexanone	5394.79±94.48	5252.48±112.48	5562.34±272.84	—	—	—	—	—	—

Pentane	65.45±6.83	4500.58±92.48	1625.65±268.37	—	—	—	48.14±21.56	3663.35±976.26	—
Nonane	31.59±1.58	3535.37±66.37	8478.74±548.27	415.15±39.36	351.34±42.45	24.71±8.26	—	2226.16±536.26	—
2-cyclopropyl butane	—	4262.38±109.36	3466.83±277.78	—	—	—	—	36.85±9.36	—
1,3,5-cycloheptene	—	4626.77±89.26	5566.53±268.27	—	—	—	1532.93±203.56	—	3556.86±698.46
1-iodo-3-methylbenzene	—	4628.88±83.48	—	—	—	—	—	—	—
octane	—	—	—	153.07±54.23	195.81±42.56	21.54±5.29	—	2462.51±596.36	28.33±7.46
toluene	—	—	—	—	—	—	99.35±27.45	2156.36±406.28	1096.84±302.55
2-furan	—	—	—	—	—	—	—	2245.75±384.66	1288.86±297.35

Table S5. The RDP classifications of the sequence reads at the phylum level. The data were expressed as the mean ± SD, n=8.

Taxonomy	con	HD	LD
Firmicutes	57.76±3.97	54.91±3.84	60.64±2.43
Bacteroidetes	17.93±3.11	30.95±2.36	22.93±1.68
Spirochaetes	18.58±2.34	1.69±0.38	9.27±2.22
Proteobacteria	2.18±0.86	3.84±0.6	4.32±1.01
Fibrobacteres	0.13±0.05	0.17±0.04	0.01±0.01
Lentisphaerae	0.44±0.16	0.23±0.03	0.01±0.01
Synergistetes	0.07±0.02	0.29±0.3	0±0
Tenericutes	0.36±0.03	0.56±0.03	0.05±0.03
Verrucomicrobia	0.04±0.02	0.1±0.04	0.01±0

Table S6. The RDP classifications of the sequence reads at the genus level in mice. The data were expressed as the mean ± SD, n=8.

Taxonomy	con	HD	LD
Prevotella	4.39±0.74	7.23±1.33	5.74±1.42
Ruminococcus	22.5±2.4	30.57±3.68	21.59±2.43
Treponema	20.58±1.52	5.81±2.39	1.99±0.59
Alistipes	5.06±1.21	3.54±0.73	2.67±0.3
Faecalibacterium	0.98±0.29	4.46±0.77	3.54±0.32
Clostridium IV	0.55±0.09	3.76±0.54	0.33±0.22
Tannerella	3.98±0.44	7.24±0.85	3.51±0.43
Gemmiger	3.74±0.76	0.08±0.01	0.8±0.31
Parabacteroides	1.38±0.3	3.85±0.82	3.43±0.33
Phascolarctobacterium	2.91±0.42	3.49±1.33	1.67±0.59
Succinivibrio	2.61±0.48	1.73±0.16	0.92±0.07
Coprococcus	1.26±0.21	4.09±0.67	2.29±0.83
Oscillibacter	0.25±0.05	2.76±0.61	1.46±0.24
Bacteroides	1.73±0.6	2.76±1.24	0.07±0.03
Paraprevotella	0.81±0.27	2.72±0.91	1.16±0.21
Barnesiella	1.52±0.37	1.28±0.2	14.49±2.33
Lactobacillus	1.28±0.36	7.43±0.91	3.58±0.73
Clostridium sensu stricto	2.61±1.07	0.7±0.17	0.74±0.23

Table S7. Distribution of 16 key genus identified by difference analysis.

Taxonomy	con	HD	LD
f__Lactobacillaceae; g__*Lactobacillus*	0.01±0.0004	0.11±0.10	0.08±0.01
f__Porphyromonadaceae; g__unclassified	0.04±0.003	0.06±0.01	0.04±0.004
f__Rikenellaceae; g__*Alistipes*	0.07±0.03	0.11±0.09	0.02±0.005
f__Ruminococcaceae; g__*Ruminococcus*	0.01±0.0009	0.05±0.009	0.02±0.01
f__Acidaminococcaceae; g__*Phascolarctobacterium*	0.01±0.01	0.05±0.03	0.02±0.004
f__Prevotellaceae; g__*Prevotella*	0.02±0.02	0.08±0.03	0.06±0.02
f__Fusobacteriaceae; g__*Fusobacterium*	0.03±0.004	0.01±0.009	0.01±0.0004
f__Porphyromonadaceae; g__*Parabacteroides*	0.009±0.002	0.04±0.01	0.03±0.002
f__Porphyromonadaceae; g__*Barnesiella*	0.01±0.001	0.008±0.005	0.13±0.02
f__Bacteroidaceae; g__*Bacteroides*	0.001±0.0005	0.03±0.02	0.009±0.008
f__Bifidobacteriaceae; g__*Bifidobacterium*	0.0001±0.00008	0.06±0.02	0.00006±0.00002
f__Ruminococcaceae; g__*Oscillibacter*	0.007±0.002	0.02±0.003	0.01±0.003
f__Ruminococcaceae; g__*Clostridium IV*	0.004±0.002	0.04±0.007	0.003±0.0003
f__Lachnospiraceae; g__*Coprococcus*	0.007±0.005	0.04±0.004	0.02±0.003
f__Ruminococcaceae; g__*Faecalibacterium*	0.007±0.005	0.05±0.02	0.03±0.003
Other	0.18±0.01	0.19±0.02	0.17±0.02
